# Behavior disparities towards blood donation in Sikkim, India

**DOI:** 10.4103/0973-6247.42692

**Published:** 2008-07

**Authors:** Namgay Shenga, Ranabir Pal, Subhabrata Sengupta

**Affiliations:** *Programme Officer, IDD cell, Govt. of Sikkim, India*; 1 *Associate Professor, Department of Community Medicine, Sikkim-Manipal Institute of Medical Sciences (SMIMS) and Central Referral Hospital (CRH), 5^th^ Mile Tadong, Gangtok, Sikkim-737 102, India*; 2 *Assistant Professor, Department of Oto- Rhino-Laryngology, Sikkim-Manipal Institute of Medical Sciences (SMIMS), Gangtok, Sikkim, India*

**Keywords:** Attitude, education, ever donors

## Abstract

**Background:**

The aim of the current research was to determine disparities in blood donation motives among the general mass of Sikkim.

**Aims:**

To identify the reasons for people donating and not donating blood voluntarily.

**Settings and Design:**

Population based cross-sectional study in Gangtok, East Sikkim.

**Materials and Methods:**

PARTICIPANTS: 300 adults by two-stage cluster sampling technique. INTERVENTIONS: None. MAIN OUTCOME MEASURES: Correlates of attitudes towards blood donation. DATA COLLECTION PROCEDURE: The data collection tool used for the study was a pre-tested structured interview schedule by which the principal investigator collected the data using interview technique.

**Statistical Analysis Used:**

Percentages and ODDS ratio were used in this study.

**Results and Conclusions:**

Out of 300 respondents, overwhelming majority (78.7%) of the respondents in the present study felt that people donate blood to save a friend or a relative. On the contrary, minority respondents (46%) were ready to donate blood voluntarily. Only 12.7% of the respondents had ever donated blood while 87.3% had never donated. Among ever donors, gender wise men donors were found to be more; 89% were married, half were from the 30 to 39 years age group. As the per-capita income or level of education increased, so did the percent of blood donors.

## Introduction

Blood has always held mysterious fascination for all and is considered to be the living force of our body. Ancient Egyptians recognized the life giving properties of blood and they used it for baths to resuscitate the sick, rejuvenate the old and infirm, and as a tonic for the treatment of various disorders. Today, the use of whole blood is a well-accepted and commonly employed measure without which many modern surgical procedures could not be carried out.[1] Human blood is an essential element of human life and there are no substitutes.[2] Safe blood is a critical component in improving health care and in preventing the spread of infectious diseases globally. Millions of lives are saved each year through blood transfusions, yet the quality and safety of blood transfusion is still a concern particularly in the developing countries. About 5% to 10% of new HIV infections worldwide are transmitted through unsafe blood transfusions. The reason for this includes blood collection from unsafe donors, poor laboratory procedures and inadequate testing of blood. Blood will be safe if there is a nationally coordinated blood transfusion service, collection of blood only from voluntary non-remunerated donors, testing of blood for transfusion transmissible infection and by transfusion of the right blood to the right patient through the appropriate clinical use of blood.[3] Nearly 7% of AIDS patients that reported to the National AIDS Program in India acquired infection following transfusion of infected blood and blood products.[4]

Sikkim is a landlocked state bounded on the north and northeast by Tibet, on the east by Bhutan, on the west by Nepal and on the south by Darjeeling district of West Bengal covering 7,096 square kilometers and a population of 540,493 as per Census 2001.[5] As per the report on screening of blood for HIV for the year 1990 to 2000 in Sikkim, 16 blood units were HIV reactive out of 9069 units screened. All these reactive units were from replacement donors.[6] There should be enough blood units in a blood bank available for everybody's requirement. But non-availability of sufficient blood units is a problem in Sikkim. The hospitals rely on the relatives of a patient to donate the necessary blood, as there are not enough voluntary blood donations to help the needy patients. The blood is donated maximum on the replacement basis. Blood banks keep their pressure on doctors, nurses and the relatives of the patient and urge them to send replacement donors to maintain their stock. This is not a good system as the relatives of the patients are pressurized to find donors and it is observed many times that professional blood donors are brought to donate blood in guise of being replacement donors. And it is a well established fact that professional donors constitute a group with high risk behavior leading to greater chances of transfusion transmitted diseases, as these donors tend to lie about their health and past medical history. The voluntary blood donation system in the state of Sikkim is 15 %.[7]

The need for blood is growing day by day as a result of advancement in the clinical medicine. In terms of the need for blood transfusion, it is noted that in the country, the death toll for road accidents has increased due to unavailability of blood transfusion services near the accident site.[8] Voluntary non-remunerated blood donation has been universally shown to be the cornerstone of safe blood.[9] The ability to transfuse blood and its separated components represent one of the great advances of modern medicine. It has made much of today's surgery possible and safer, and it has saved and prolonged countless lives in war and in peace. But it is not without perils. Every blood transfusion carries with it some calculated risk. It is not now and probably never will be completely safe.[10] Truly speaking voluntary blood donors are the bricks of the edifice called blood transfusion. The presence of professional blood sellers, however, cannot be ruled out among the replacement donor in the garb of relatives or friends. This fact is supported by the WHO report, which says, “All the countries admitted that the absences of paid blood sellers are not real. They exist in replacement donor category.[11]

The aim of the current research was to determine the disparities in blood donation behavior among the present donors and investigate their attitude towards donation to understand the problems and to improve voluntary blood donation at Gangtok, East Sikkim. To the horizon of our knowledge there had been no study done in this field in the state of Sikkim and this was one of the first studies from North East part of India.

## Matarials and Methods

There were 15 polling stations under the Gangtok Assembly constituency with a total of 12199 voters. Each polling station had a total of 500 to 1200 voters. In the first stage, 10 polling stations were selected randomly through draw of lots. In the second stage, a total of thirty adults were selected from each polling station randomly.

The house number was matched with the name and the serial number of the adult subjects. The house number was traced and then the subject randomly selected was identified for the findings of the study. The permission to conduct the study in Gangtok and STNM Hospital was taken from the office of the Secretary Health and Family Welfare Department, Government of Sikkim. All the participants were explained about the purpose of the study and were ensured strict confidentiality, and then verbal informed consent was taken from each of them before the interview. The participants were given the options not to participate in the study if they wanted. Then by interview technique the principal investigator Dr. Namgay Shenga collected the data. The collection of the data was from the 15^th^ of January till the 30^th^ of March 2004. On an average 5 to 6 interviews were conducted in a day. Details of the questionnaire can be provided, if required. Information on blood donation was disseminated in health education sessions to complement the findings of study.

### Statistical analysis used

The data collected were thoroughly cleaned and entered into Excel spread sheets and analysis was carried out using SPSS, version 11 software. The procedures involved were transcription, preliminary data inspection, content analysis and interpretation. Percentages and ODDS ratio were used in this study to find association of attitude of attitude correlates with voluntary blood donation.

## Results

The study was designed to assess the general attitude of the study participants to understand, why people donate blood. The respondents were given multiple options for this question. Overwhelming majority (78.7%) of the respondents in the present study felt that people donate blood to save a friend or a relative; 15% responded that people donate blood to get healthy; 3.7% responded that they do it to get some free investigations done for diseases like HIV/AIDS and Hepatitis and 2.7% responded that they donate for money [Table 1].

**Table 1:** Attitude correlates of blood donation

Statement	Number	Percentage
People donate blood to save a relative or a friend	236	78.7
Donating blood makes one healthy	45	15.0
People donate blood to get some money	8	2.7
People donate blood to get some free investigations done	11	3.7
Total	300	100

Incidentally, the next question was precisely about their attitude on intended practice towards voluntary blood donation. The positive respondents came down to minority (46%), who were ready to donate blood voluntarily. 31.7% percent of the participants showed negative attitude towards voluntary blood donation while 22.3% were yet undecided [Table 2]. Next question followed was more precise about their personal contribution of blood donation history. Out of 300 subjects, only 12.7% of the respondents had ever donated blood, while 87.3% had never donated in their life-time [Table 3]. Now, in-depth study was done on the characteristics of these 38 ever donors. Gender-wise greater proportion of the blood donors were men than the women counterparts in our sample. Eighty nine percent of them were married and 50% of them were employed. Almost similar percent donors were from both Hindu and Buddhist religion groups, while the schedule tribe was the highest among the community status group. These donors were mostly educated belonging to the higher income group. Age-wise the maximum donors were from the 30 to 39 years age group [Table 4]. In our study, as the per-capita income or level of education increased, so did the percent of blood donors in the population. It was found that odds of having a positive attitude towards voluntary blood donation is 1.5 times more in those with better educational levels then those with lesser educational levels. (OR 1.5; 95% CI 1.2 – 1.9) [Table 5].

**Table 2:** Attitude on intended practice towards voluntary blood donation

Donate blood voluntarily?	Number	Percentage
Yes	138	46.0
No	95	31.7
Can't say	67	22.3
Total	300	100

**Table 3:** Practice towards blood donation

Ever donated blood?	Number	Percentage
Yes	38	12.7
No	262	87.3
Total	300	100

**Table 4:** Characteristics of the ever donors (n=38)

Variables	Ever donors (%)
Sex	
Male	32 (84.2)
Female	6 (15.8)
Marital status	
Married	34 (89.5)
Unmarried	4 (10.5)
Occupation	
Unemployed	6 (15.8)
Office goers	19 (50.0)
Businessman	6 (15.8)
Others	7 (18.4)
Religion	
Hindu	17 (44.7)
Buddhist	17 (44.7)
Others	4 (10.3)
Community status	
Schedule tribe	16 (42.1)
Schedule caste	1 (2.6)
Other backward classes	8 (21.1)
Others	13 (34.2)
Age group	
<30 years	8 (21.1)
30 to 39 years	19 (50.0)
> =40	11(28.9)

**Table 5:** Characteristics of the ever donors (n=38)

Variables	Ever donors (%)
Education	
Illiterate	Nil
Primary school completed	1 (2.6)
Junior school completed	9 (23.7)
Secondary school completed	11 (28.9)
Higher secondary and above	17 (44.7)
Per-capita monthly income	
<700	1 (2.6)
700 – 1100	12 (31.6)
> 1100	25 (65.8)

## Discussion

In Sikkim, health care services are provided by the government health services network even in the remotest corner. There is no private nursing home in the state. ‘Sikkim Manipal Institute of Medical Sciences’ has been started in the state as the only medical college in this state of Sikkim as public-private partnership. The state is having three licensed Blood Banks - one at the state level the Central Blood Bank at STNM Hospital Gangtok, one in Sikkim Manipal Institute of Medical Sciences, Gangtok and one at the district Level the Blood Bank at Namchi, south Sikkim Specialized medical and surgical services are available at these three centers. Hence, these centers are provided with blood transfusion services. There is no facility for preparation of blood components or fractionation of blood in the state. Our observation regarding disparity of attitude and reported practice was that, of the total 300 participants, 12.7% had donated blood. These donors were mostly educated, employed, married and from higher income group.

Comparable observations were also found among the students of Chulalongkon University, Thailand, where 80% of the participants knew about blood donation and 11% of the study population (n =400) had even donated blood voluntarily. The study, however, did not find any significant correlation of gender, age, and educational level with knowledge about blood donation. The findings of the study concluded that greater knowledge about blood donation does not lead to donation and specific campaigns are needed to convert this into actual voluntary donation.[12]

Another similar study among the students of University of Dhaka, Bangladesh showed that 80% of the participants showed a positive attitude towards blood donation; however, only 16% of the respondents in this study (n = 200) had actually ever donated blood voluntarily. Physical harm and fear were found to be the common reasons for not donating blood. The results also showed that a high number of respondents had a negative attitude towards blood donation.[13] A study in Delhi urban slum reported that 7.7% of the participants (n =434) were ever donors.[14] Similar reasons were found in Australia in a study among the college students. The reluctance was mostly due to fear, possible illness and inconvenience of giving blood.[15] Another study in Mexico also found that non-donation was mainly due to the fear of getting dizzy at the site of blood.[16]

A study in Lagos Nigeria also found that 47% were afraid to donate because of the possible side-effects like weight loss, sexual failure, high blood pressure, sudden death and convulsions. It was also found in the same study that 41% had preferred certificate as an incentive, 13.65% preferred money and 2.58% would donate for nothing.[17] Comparatively, in the present study 49% of the participants showed willingness to donate blood for a future assurance of free blood for the donor or his direct family, while 20.3% preferred some kind of non-monetary benefit in the form of certificates of recognition or time off from the office; only 4.3% preferred some monetary benefit and 25.3% did not want anything. This finding indicates that an incentive can be used in enhancing the effectiveness of blood donation campaigns.

A study in Baltimore also found that the donors would be encouraged to donate if specific incentives were offered. The highest response was for future blood credits and medical testing.[18] Another study in Texas also concluded that individuals donate in order to reduce medical risks and that earning future blood credits would be a primary motivator.[19] A study amongst the adults in Mwanza Region, Tanzania also noted a positive attitude towards voluntary blood donation but majority of the people will do so only for an incentive.[20] The current results are in agreement with one study in United States where it was found that educational level and family income are both strong indicators of the probability of someone's donating blood.[21]

### Limitations of the study

This study was done as part of Masters of Public Health programme as detailed in ‘METHODS’ at Achutha Menon Centre for Health Science Studies, Sree Chitra Tirunal Institute for Medical Sciences and Technology, Thiruvananthapuram, Kerala, India. The study could not be developed and followed-up later to understand the reason behind this disparity as the native state of the principal investigator Dr. Namgay Shenga was long way from where the study was initiated.

### Recommendations

Donation system in the state. Voluntary donation system is by far the best and it needs to be strengthened. Thus, the study of understanding the various factors that could change the perception and awareness about blood donation among the general population may come out to be useful for the successful implementation of blood donation program in the state, especially in improving the voluntary blood donation system. India is a multi-cultural multi-lingual and geographically uneven country. Any short term solution may not help us to reach the goal of universal voluntary blood donation in India in near future. The health professionals alone will not be able to meet this mammoth task.

Given the findings in the present study, the following recommendations are made:

#### Health education

Health education system needs to improve knowledge about blood donation among the people with lesser educational level. It could be done by means of improving educational tools preferably based on audiovisual techniques. Preferably school curriculum may include materials to allay fears hovering around voluntary blood donation. The system should create wider awareness about the importance of voluntary blood donation and encourage more people to become regular donors.

#### Removal of myths and misconceptions

The information education and communication system should have some productive advertisements to motivate the general public for voluntary blood donation. The advertisements need to address the fear factor, which is of great concern to all the blood donors. It also must focus on clearing the myths and misconceptions about blood donations and keep the people well informed about the importance of saving life through blood donation.

#### Provision of incentives

The general public must be encouraged and motivated to donate blood. There should be provision of incentives like future assurance of free blood for the donor or his wards/ relations/ friends. A blood insurance scheme may be started. A non-monetary incentive in the form of certificates of recognition may also be another motivating factor to improve voluntary blood donation.

#### Provision of better facilities

Provision of better facilities in a blood bank, spreading awareness about the advantages of blood donation not only for the recipient but also for the donor himself could be a motivating factor. Making people aware of recent findings, like frequent and long-term blood donation is associated with a lower risk of cardiovascular events in men can motivate population to be regular donors.

## Conclusions

The finding of the research indicated that there lies an ocean of difference between the attitude towards voluntary blood donation and that of true life practice among general mass of the capital of this north-eastern hilly state of India. Proper selection of the donor is the more important even in replacement and relative donors. We should spend some extra time in case of counseling of close associates and relatives of the recipient. The regular flow of relative donor will have a cumulative effect on the different strata of society leading to reduction in unnecessary fear associated with voluntary blood donation. We have to sincerely enquire into the family history separately to motivate them in a socio-cultural setting both inside the hospital as well as in the community. We can even get safe blood from relative donor provided proper selection of donor and we should highlight it.
